# Hepatotoxicity by Dietary Supplements: A Tabular Listing and Clinical Characteristics

**DOI:** 10.3390/ijms17040537

**Published:** 2016-04-09

**Authors:** Miren García-Cortés, Mercedes Robles-Díaz, Aida Ortega-Alonso, Inmaculada Medina-Caliz, Raul J. Andrade

**Affiliations:** 1 Servicio de Farmacología Clíınica and Unidad de Gestión Clínica (UGC) de Gastroenterología y Hepatología, Instituto de Investigación Biomédica de Málaga (IBIMA), Hospital Universitario Virgen de la Victoria, Universidad de Málaga (UMA), 29010 Málaga, Spain; mirengar1@hotmail.com (M.G.-C.); aida_ortega_alonso@hotmail.com (A.O.-A.); imcaliz@uma.es (I.M.-C.); andrade@uma.es (R.J.A.); 2 Centro de Investigación Biomédica en Red de Enfermedades Hepáticas y Digestivas (CIBERehd), 28029 Madrid, Spain

**Keywords:** dietary supplements, liver injury, hepatotoxicity, anabolic steroids, green tea, Herbalife products, Hydroxycut, Oxyelite Pro, vitamin A, usnic acid

## Abstract

Dietary supplements (DS) are extensively consumed worldwide despite unproven efficacy. The true incidence of DS-induced liver injury (DSILI) is unknown but is probably under-diagnosed due to the general belief of safety of these products. Reported cases of herbals and DS-induced liver injury are increasing worldwide. The aim of this manuscript is to report a tabular listing with a description of DS associated with hepatotoxicity as well as review the phenotype and severity of DSILI. Natural remedies related to hepatotoxicity can be divided into herbal product-induced liver injury and DS-induced liver injury. In this article, we describe different DS associated with liver injury, some of them manufactured DS containing several ingredients (Herbalife™ products, Hydroxycut™, LipoKinetix™, UCP-1 and OxyELITE™) while others have a single ingredient (green tea extract, linoleic acid, usnic acid, 1,3-Dimethylamylamine, vitamin A, *Garcinia cambogia* and ma huang). Additional DS containing some of the aforementioned ingredients implicated in liver injury are also covered. We have also included illicit androgenic anabolic steroids for bodybuilding in this work, as they are frequently sold under the denomination of DS despite being conventional drugs.

## 1. Introduction

Herbals and dietary supplements (HDS) are used to maintain or improve health. Regulation of herbal products may vary between different countries. In the European Union, the concepts of traditional herbal medicines and traditional plant food supplements are defined under different legal frameworks [1]. The European Directive 2004/24/EC released in 2004 by the European Parliament and by the European Council provides the guidelines for the use of herbal medicines; where an herbal product is considered a medicinal product when presented as having properties for treating or preventing disease in human beings or when it has a pharmacological, immunological or metabolic action. It is the competence and responsibility of national authorities to decide, on a case-by-case basis, whether an herbal product fulfils the definition of medicinal product. On the other hand, herbal products marketed in the form of food supplements should comply with Directive 2002/46/EC on food supplements and Regulation (EC) No 1924/2006 on nutrition and health claims made on foods of the European Food Safety Authority (EFSA) (http://www.efsa.europa.eu/). An herb may be considered a medicinal product or a dietary supplement (DS) depending on medical claims of a therapeutic indication [2]. In the United States (US), the Dietary Supplement Health and Education Act (DSHEA) of 1994 remains the foundation for current regulation of herbal products that are all classified as DS or botanicals [3]. Therefore, they are regulated as food products and not subjected to the same premarket requirements for safety or efficacy.

Although the real prevalence of HDS consumption is unknown, it is estimated that more than 50% of the US adult population uses HDS [4,5]. Besides, a recent survey made in Europe (Finland, Germany, Romania, Italy, Spain, and the United Kingdom (UK)) estimated that 18.8% out of 2359 consumers admitted using one or more DS, excluding herbal products [6].

However, HDS are not as safe as many people believe. These products can induce adverse effects including liver injury. Moreover, occurrence of HDS-related liver toxicity ranges from 2% to 16% of all identified cases of hepatotoxicity included in different drug-induced liver injury (DILI) Registries in Western countries with an increasing prevalence over time in the US [7,8,9,10]. Besides, the number of illicit anabolic androgenic steroids (AAS) inducing liver injury and submitted to the DILI Network (DILIN) in the US and to the Spanish DILI Registry has also increased in recent years [10,11]. An even higher prevalence of HDS-DILI can be found in Asian countries where there is a widespread consumption of HDS, 73% in Korea, 71% in Singapore, and 40% in China [12,13,14].

Unlike what happens with conventional drugs that have the Anatomical Therapeutic Chemical (ATC) classification system, an unresolved problem is the lack of a standard nomenclature or classification scheme for HDS. Stickel *et al.* classified HDS related to hepatotoxicity into two different groups: herbal-induced liver injury and dietary supplement induced liver injury (DSILI) [15]. This author considers DS if consumed as an aid to improve nutritional status, to lose weight or to treat constipation [15]. The DSHEA defines DS as any product intended to supplement, but not substitute the diet. Dietary supplements may contain one or more ingredients including vitamins, minerals, herbs, botanicals, aminoacids or extracts thereof [3]. Although herbals and DS sometimes overlap, the substances considered as DS associated with liver injury are *Camellia sinensis* (green tea extract), usnic acid, 1,3-Dimethylamylamine (1,3-DMAA), vitamin A, Herbalife products, Hydroxycut, LipoKinetix, UCP-1, OxyELITE pro, and anabolic androgenic steroids [16,17]. We also include in this list linoleic acid, ma huang and *Garcinia cambogia*, DS marketed for weight loss.

DSILI is challenging due to the fact that these products are not regulated in the same way as prescription drugs are, and subsequently lack uniform criteria for manufacturing and authentication of this products. Probably, underreporting is even higher with DS than with DILI given that consumers and health care practitioners are not always aware of the possible adverse events of these supplements. Moreover, as occurs with DILI, diagnosis and causality assessment of DSILI is difficult given the absence of diagnostic tests or biomarkers, and causality assessment methods such as the Roussel Uclaf Causality Assessment Method (RUCAM) [18] are not completely validated for this purpose. An attempt to develop a specific scale for the causality assessment of HDS-DILI was made by the DILIN group, but it has not been validated and published [19].

In this manuscript we aimed to make a tabular listing with a detailed description of potentially hepatotoxic DS as well as review the phenotype and severity of DSILI. Herbal products have been described elsewhere in other chapters of this special issue. A selective literature search in the PubMed was performed, using search term such as dietary supplements combined with the following: drug-induced liver injury, herb-induced liver injury, hepatotoxicity, liver damage, and hepatitis. Besides, an individual research was made for each DS related to hepatotoxicity. The search was primarily focused on English and Spanish language case reports, case series, and clinical reviews, published from 1984 to November 2015. All citations in these reports were searched for other yet unidentified case reports, including cases with sufficient information published in other languages.

## 2. Reported Cases of Dietary Supplements-Induced Liver Injury: A Tabular Listing

From overall publications, different DS were identified as being linked to hepatotoxicity, green tea extracts, linoleic acid, usnic acid, vitamin A, garcinia cambogia, ma huang, 1,3-DMAA and multi-ingredient products such as Herbalife products, Hydroxycut, LipoKinetix, Oxy ELITE Pro and UCP-1 and anabolic steroids illicitly sold as DS for bodybuilding. Anabolic androgenic steroids can be divided into two groups: legal AAS with a prescription from a physician for a medical condition and new designer steroids (“underground” drugs) that are not used clinically and are entirely illegal. Although strictly speaking none of them are DS, we have included illicit AAS in this manuscript because many of them are sold under the description of DS despite being conventional drugs (ATC classification system A14A anabolic steroids). Table 1 summarizes information retrieved from original case reports and cases from DILI registries associated with the aforementioned DS.

### 2.1. Illiciti Androgenic Anabolic Steroids for Body Building

Anabolic androgenic steroids are synthetic derivatives of testosterone whose medical indications are mainly male hypogonadism, breast cancer, anemia and hereditary angioneurotic edema. However, several AAS such as stanozolol, methyltestosterone, oxandrolone, fluoxymesterone and danazol are also used without medical supervision for performance enhancement and muscle building purposes due to their anabolic effects, stimulating protein synthesis and positive nitrogen balance [20].

There are numerous reported cases of liver injury due to bodybuilding products, most of them containing illicit anabolic steroids [10,11,21,22,23]. Among the most frequent AAS involved in liver injury are stanozolol, methasterone, methylepithiostanol [11,24,25,26]. The number of anabolic androgenic steroids (AAS) hepatotoxicity cases submitted to the Spanish DILI Registry has increased in recent years. Only five AAS hepatotoxicity cases were identified during the first 15 years (1994–2009), while the number of cases tripled to 15 over the next four years (2010–2013) resulting in a significant increase from 1% to 8% of the total number of hepatotoxicity cases in the Spanish DILI Registry [11]. The reason for this increase could be a combination of increased usage and improved clinical awareness of this form of hepatotoxicity. Similarly, the Drug–Induced Liver Injury Network found an increase in bodybuilding HDS cases from 2% in 2004-2005 to 8% in 2010–2012 [10].

Several patterns of injury such as focal nodular hyperplasia, hepatocellular adenoma [27], hepatocellular carcinoma [28], peliosis hepatis [29,30], spontaneous hepatic rupture [31] and, specially, cholestasis hepatitis [11,24,25,26,32,33,34,35,36], have been described. However, in a recent study, AAS hepatotoxicity was associated with a distinct phenotype, characterized by considerable total bilirubin (TB) elevations independent of type of damage, in addition to low values of transaminases and alkaline phosphatase, compared to values in liver injury due to convencional drugs and herbs [10,11,24,25,26,37].

Young men (mean age 32 years), requiring hospitalisation, hepatocellular injury and jaundice were predominating features among the AAS cases in the Spanish and SLatin DILI Registries. Although hepatocellular injury predominates in this study, acute kidney injury developed in cholestatic cases with pronounced jaundice [11]. The precise mechanism of toxicity is not clear, but the genotyping of patients with cholestatic pattern of liver injury due to anabolic steroids suggests that these products could induce inhibition of biliary transporter proteins such as ATP8B1/ABCB11, as ocurr in cases of benign recurrent intrahepatic cholestasis type 1 or 2 [38].

A study performed in 2011 in Brazil, suggested that anabolic steroids could induce non-alcoholic fatty liver disease (NAFLD). Comparing two groups of bodybuilders, one consuming AAS with another that did not, 12.6% had criteria of NAFLD (compatible imagen on liver ultrasound, elevated transaminases, and exclusion of overweight, insulin resistance, significant alcohol consume or other medication that could be related with NAFLD) *vs.* 2.4%, respectively [39]. In 2015, another Brazilian study with 182 asymptomatic young recreational AAS using bodybuilders was performed. They, all ≥18 years old and with AAS use for ≥6 months, presented a wide spectrum of liver injuries that included hepatotoxicity, fatty liver, and liver neoplasm [27].

In addition to liver injury, many other effects have been described in AAS users: cardiovascular effects (hypertension, cardiomyopathy, left ventricular hypertrophy, dyslipidemia with potential acceleration of aterosclerosis, myocardial ischemia, adverse effects on coagulation and platelet aggregation, and arrhythmias); neuroendocrine effects (temporarily hypogonadism after stopping a cycle of AAS due to the suppression of the hypothalamic-pituitary-testicular axis); and neuropsychiatric effects (hypomanic or manic symptoms, sometimes associated with aggression and violence, although it may be difficult to judge which of these psychiatric effects are attributable to AAS themselves, as opposed to underlying personality or psychosocial factors surrounding AAS use) [40].

### 2.2. Green Tea (Camellia sinensis) Extracts

Green tea is a highly consumed and popular drink in the world used for centuries. Depending on the processing treatment, especially the “degree of fermentation”, tea can obtain differences in flavor, color and composition. Green tea contains methylxanthine alkaloids (caffeine, theophylline, theobromine), the polyphenols which are considered the major bioactive molecules of tea. In the last decade, the consumption of tea has increased for its health benefits. While green tea infusion is widely consumed and generally safe (although some cases of liver injury after green tea infusion intake have been reported, Table 1), green tea extracts have shown to have a hepatotoxic potential. The first report on liver damage related to green tea extract intake was in 1999 [41]. Thereafter, many cases of liver injury related to the intake of different green tea extracts have been recorded [8,15,16,42,43,44,45,46,47,48,49,50,51,52]. Published cases of hepatotoxicity due to green tea extracts from the US, UK and Australia until 2008 were reviewed by the US Pharmacopeia. Out of 34 evaluated cases, 27 were labelled as probably and the remaining 7 cases as possibly induced by green tea extracts. One of these patients died, which indicate that this kind of hepatotoxicity can have serious outcomes [53]. In a review performed by Mazzantion 34 published cases and two unpublished, seven had a positive rechallenge due to green tea extracts [54]. This highlight the importance of a correct hepatotoxicity diagnosis to avoid inadvertent reexposition to the causative agent.

The physiopathology of green tea inducing liver damage is unclear but could be explained by the (−)-epigallocatechin gallate or its metabolite (−)-epicatechin gallate which, in certain conditions such as fasting, can induce oxidative stress and liver damage [55]. However, experimental studies have also demonstrated hepatoprotective properties *in vitro* and *in vivo* [56,57,58]. Furtheremore, a recent systematic review presents therapeutic and favorable effects of *Camellia sinensis* in humans, such as reducing mortality, attenuating steatosis, and a reducing incidence of primary liver cancer [59].

The clinical presentation and liver profile of three hepatotoxicity cases induced by *Camellia sinensis* extracts from the Spanish DILI Registry includes hepatocellular pattern of liver injury, jaundice with total bilirubin higher than 10 times the upper limit of normal (× ULN) and high level of alanine aminotransferase (ALT) (>45× ULN). The duration of treatment and time to onset range from 17 to 121 days and 5 to 121 days, respectively [8]. Five hepatotoxicity cases due to Cuur (*Camellia sinensis*) reported to the Swedish Adverse Drug Reactions Advisory Committee showed a similar profile with hepatocellular pattern of liver injury, and four with jaundice and high levels of ALT (25–95× ULN) and duration of treatment from 35 to 140 days [47]. Another case of hepatotoxicity was reported in Spain in 2004 in a woman who had taken Camilina-Arkocápsulas (*Camelia sinensis*) and Ortosifón Arkocápsulas (*Orthosiphon stamineus*) for weigh loss for two months. Similarly, hepatocellular pattern of liver damage with high level of ALT (2398 U/L) and TB (19.9 mg/dL) was found [49]. Fulminant hepatitis that required liver transplantation has also been associated with Exolise, an 80% ethanolic dry extract of green tea (*Camellia sinensis*) standardized at 25% catechins expressed as epigallocatechin gallate, containing 5%–10% caffeine [60]. Five cases of liver injury due to Exolise had been reported in Spain before this fulminant case [50,61]. In fact, Exolise was withdrawal in Spain in 2003 (Spanish Drug and Sanitary Products Agency, AEMPS).

There are some cases of liver injury due to green tea extracts alone but in many cases the patients also took other drugs or products with potential hepatotoxicity, such as seen in a 28-year-old woman taking Somalyz (usnic acid, propionyl-l-carnitine, phosphatidylcholine/phosphatidylethanolamine, gamma-aminobutyric acid, and vitamin E) and Lipolyz (usnic acid, propionyl-l-carnitine, green tea extract, guggulsterone Z and guggulster-one E, cyclic adenosine monophosphate and vitamin E) for body building purposes. Liver test showed TB 4× ULN, ALT 23× ULN, aspartate aminotransferase (AST) 11.6× ULN, alkaline phosphatase (ALP) 1× ULN and international normalized ratio (INR) 2.6. The patient developed encephalopathy and required liver transplantation [62].

Despite the difficulty in identifying green tea extracts as the culprit in an episode of liver damage due to comcomitant intake of other products, as well as the assumption of green tea being a safe product, structured causality assessments have suggested causal relationships between intake of green tea extracts and liver damage with cases of positive reexposures as mentioned above [54,63].

### 2.3. Linoleic Acid

Conjugated linoleic acid (CLA), a polyunsaturated omega-6 fatty acid is a DS that has been shown to cause reduction in body fat mass. It has also been shown to stimulate immune responses, improve insuline sensitivity, and modify lipid metabolism. Despite the benefits attributed to CLA, three cases of drug-induced liver injury due to CLA have been reported to date, the first one in 2009 [64]. All patients were women and developed hepatocelular type of liver injury after taking CLA to aid in weight loss and body fat reduction. The most severe case was a 63-year-old female who had taken purely CLA pills during one month, and presented at admission high levels of ALT (2300 U/L), AST (2300 U/L), TB (26 mg/dL), ALP (255 U/L) and INR 1.65. The patient developed encephalopathy and required liver transplantation [65]. Out of the other two patients, one presented jaundice (TB 12.9 mg/dL) [64], while both presented elevated values of transaminases at onset (ALT 2101 U/L, AST 1663 U/L and ALT 1078 U/L, AST 1519 U/L, respectively) [64,66]. Both cases had complete resolution after discontinuation of CLA.

### 2.4. Usnic Acid, LipoKinetix and UCP-1

Usnic acid is uniquely found in lichens, and is especially abundant in genera of lichens such as *Alectoria*, *Cladonia*, *Usnea*, *Lecanora*, *Ramalina* and *Evernia*. Many lichens and extracts containing usnic acid have been utilized for medicinal, perfumery, cosmetic as well as ecological applications [67]. The clinical properties reported are antimicrobial, anti-inflammatory, antioxidant, analgesic, antipiretic and weight loss. The most popular indication is for weight control alone or in combination with other herbal products. Usnic acid functions as an uncoupler of the mitochondrial respiratory chain and is subsequently believed to stimulate fuel oxidation and increase metabolic rates, which could influence weight loss [68]. However, this mechanism could also induce mitochondrial injury and hepatocyte death, as *in vitro* studies have shown that uncoupling mitochondrial oxidative phosphorylation can generate oxidative stress [69].

Several cases of acute liver failure (ALF) related to a combination of DS with usnic acid alone or in combination with other products have been reported, including cases requiring liver transplantation [70,71,72,73,74]. Hepatotoxicity has been described related to the intake of a multiingredient preparation LipoKinetix, a product that contains norephedrine, caffeine, yohimbine, diiodothyronine, and sodium usniate (usnic acid) [70,73,74]. Favreau *et al.* described seven cases of acute liver injury related to LipoKinetix. Time to onset for liver injury usually occurred within the first 3 months of consumption. Pattern of liver test results were compatible with drug-induced acute hepatocellular necrosis. There was no evidence of allergy, such as rash or eosinophilia. One patient developed ALF. All recovered spontaneously after discontinuing use of LipoKinetix, and results of liver tests as well as symptoms normalized within 4 months in five patients (two patients declined to have further testing). Three of the seven patients, including the one who developed ALF, were taking LipoKinetix alone at the time of presentation. Of the four patients who were taking multiple supplements, two resumed taking supplements other than LipoKinetix without further incidents. Other causes of liver injury were excluded in all the patients [70].

In a case series of ALF from an adult tertiary care university hospital and a Veterans Affairs hospital in Oregon describing 20 cases of ALF, 10 were recent or active users of DS or herbs. In two of these cases, a 25-year-old female who died and a 42-year-old male who recovered, Lipokinetix was identified as the only cause of ALF [73].

An analysis, performed at four transplant centers, found an elevated number of acute hepatitis or ALF due to herbal products for weight loss. Out of 12 patients with liver injury attributed to the intake of HDS weight loss products, two cases in two 32-year-old females were due to Lipokinetix [74]. Hence, the US Food and Drug Administration (FDA) published a warning about LipoKinetix, and the product was withdrawn from the US market in November 2001.

Durazo *et al.* reported a case of a 28-year-old woman who developed ALF and required orthotopic liver transplantation after two weeks of intake of pure usnic acid for weight loss [71]. Another two cases of severe liver toxicity related to a multi-ingredient health supplement UCP-1 (BDC Nutrition, Richmond, KY, USA), a combination containing usnic acid, l-carnitine, and calcium pyruvate, were published by Sanchez *et al.* in 2006. They reported acute liver injury in a wife and husband after taking this product for bodybuilding purposes. The wife developed ALF that required liver transplantation [72]. A genetic susceptibility has been suggested after the report of three sisters with liver toxicity related to a “fat burner” DS containing usnic acid [75].

Finally, another case of hepatotoxicity requiring liver transplantation due to DS (Somalyz and Lipolyz, Species Nutrition, Westbury, NY, USA) containing usnic acid was reported by Yellapu *et al.* in 2011 [62]. This case, is described in the *Camellia sinensis* section as green tea extract is one of the component of Lipolyz and we are unable to determine which component is responsible for liver injury or if there is an interaction between the components that cause hepatotoxicity.

### 2.5. Herbalife Products

Herbalife is a company that produces different products for weight loss, DS and cosmetics. Products manufactured in the US are exported to more than 50 countries around the world [76]. In 2014, the company reported net sales of $5.0 billion, a 3% increase compared to 2013 [77]. Up to date, there are 11 published reports of liver injury (with a total of 57 cases) after the intake of some of the different products offered by Herbalife, 12 cases from Switzerland [78,79], 12 from Israel [80], 20 from Spain [81,82,83], 1 from Argentina [84], 5 from Iceland [85], 5 from US [10,86] and two from Venezuela [87]. The more frequent type of liver injury in these cases was hepatocellular, although there were also cases with cholestatic and mixed liver injury. Among the Herbalife products hepatotoxicity cases there were cases of acute liver failure requiring transplants and cases of chronic liver injury including cirrhosis. The mechanism of hepatotoxicity is very difficult to identify as most of the patients took several different Herbalife products at the same time. Some patients had elevated titers of autoantibodies and liver biopsies with plasma cell infiltrates, suggesting that autoimmune mechanisms could have played a role in these patients. In two cases of liver injury after intake of Herbalife products, contaminations with *Bacillus subtilis* were found in the products. Hence, adulteration of products with bacterial pathogens could explain some cases of liver injury in patients taking Herbalife products [79]. Other possible causes of liver injury could be contamination with other microorganisms or chemicals during the manufacturing process or the use of unrefined products, such as herb extracts.

### 2.6. Hydroxycut

Hydroxycut is a DS product for weight loss and muscle building with many changes in its formulation. Some of the ingredients have been *Garcinia cambogia*, *Cissus quadrangularis*, caffeine, Ma Huang (ephedra) and green tea. At least, 29 cases of Hydroxicut-induced liver injury have been reported [10,74,86,88,89,90,91,92,93,94,95,96,97,98,99]. Typically, the episodes occur after weeks of consumption and show a hepatocellular pattern of liver damage (25/28) and high levels of transaminases. Only few cases showed cholestasis (3/28). In five cases reported of Hydroxycut-induced hepatotoxicity, from a case series of 130 different HDS-induced liver injury cases, the pattern of liver injury is not specified [10]. Autoimmune markers can be positive for antinuclear antibodies at the time of the acute liver injury in some patients [92]. In these reports, six patients developed ALF; out of them, three received a liver transplantation. An additional patient underwent exploratory laparotomy for liver transplantation, and was found to have intestinal infarction. The liver transplantation was aborted and the patient died [74,92].

The formulation of Hydroxycut has changed in recent years. The earliest reported cases of acute liver injury related to Hydroxycut were part of a case series from four transplant centers of patients that had developed severe hepatitis after taking various supplements containing ephedra, also called ma huang, a plant substance whose natural ingredient is ephedrine [74]. In 2004 the sale of supplements containing ephedra was banned by the FDA. However, new subsequent cases of ma huang-free Hydroxycut-associated hepatotoxicity were reported, all of whom spontaneously recovered. In May 2009, the FDA published a warning about Hydroxycut products related to hepatotoxicity, resulting in that these products were withdrawn from the market [95]. However, after the 2009 recall, Hydroxycut was reformulated and reintroduced on the market, and FDA confirmed that the only ingredient left from prior formulations was caffeine [96]. Despite this, a new case of hepatotoxicity due to Hydroxycut SX-7 Clean Sensory formulation was reported in 2015, with high level of transaminases (AST 2360 UI/L, ALT 6218 UI/L), TB 8.3 mg/dL, alteration in coagulation (INR) 5.0) and renal impairment (creatinine 1.80 mg/dL) [97].

### 2.7. 1,3-Dimethylamylamine (DMAA) and OxyELITE

OxyELITE Pro (OEP) is a DS containing DMAA. It is used for performance enhancement and muscle building and for weight control as it is believed to accelerate metabolic processes. DMAA is an ingredient in over 20 DS and has been previously implicated in acute myocardial infarction (Jack3d) [100]. A case series of OEP-induced liver injury in a military population in Southern California with two cases requiring transplants due to acute liver failure was reported in 2014 [101].

In early 2013, several reformulated (DMAA-free) versions of OEP, in which 1,3-dimethylamylamine was replaced with aegeline, started to be sold. However, this new ingredient in OEP products was not notified to the FDA. Roytman reported, also in 2014, another case series of eight patients in Hawaii with acute liver injury after taking the new DMAA-free OEP formula “Super Thermo”. Six of the patients had also taken the “old formula” for months. Two patients underwent urgent transplantations, and one died [102]. Later, a deep epidemiological investigation of the events in Hawaii was carried out. The Hawaii Department of Health identified 36 individuals with acute onset hepatitis (including the patients from the Roytman’s case series [102]), using OEP (some of them DMAA-free formulations) 60 days before liver damage onset and residence in Hawaii during the exposure period. Fourteen patients used only OEP, while 22 subjects used OEP and another DS as well. Hepatitis A, B and C, alternative diagnosis in hepatic imaging and history of alcoholism were ruled out in all the patients. The medians of ALT, AST and TB peak were 1740 U/L, 1134 U/L and 9.4 mg/d/L, respectively. No more cases of ALF than those previously reported by Roytman were found [103]. However, a further investigation by Teschke *et al.* of the cause(s) of liver disease in this cluster of suspected OEP hepatotoxicity patients in Hawaii reached the conclusion that there was insufficient evidence to determine OEP as the culprit in all the cases [104].

Additional cases of liver injury associated with the consumption of OEP have been reported. One case of liver injury attributed to OEP was presented in a case series of hepatotoxicity due to HDS from the Drug-Induced Liver Injury Network [10]. Another study investigating 114 reports of adverse event submitted to the FDA from February 2012 to February 2014 in patients who ingested OEP was performed. Out of the 114 cases, 55 developed liver disease (viral and autoimmune hepatitis, gallbladder disease and other known causes of liver damage were excluded) [105].

### 2.8. Vitamin A

Vitamin A is used to improve immune functions, night blindness, prevent and treat vitamin A deficiency, and also for skin conditions including eczema, psoriasis, keratosis follicularis and ichthyosis. Acute toxicity due to hypervitaminosis A, occurs when adults and children ingest >100× and >20× the recommended daily allowance, respectively, for vitamin A over a period of hours or a few days [106]. It is a small problem compared to chronic vitamin A toxicity from the ingestion of high amounts of vitamin A for months or years. Daily intakes of >25,000 IU for >6 years and >100,000 IU for >6 months are considered toxic, but there is wide inter-individual variability for the lowest intake required to elicit toxicity [107,108,109]. Long-term use of large amounts of vitamin A might cause side effects including fatigue, irritability, mental changes, anorexia, stomach discomfort, nausea, vomiting, mild fever, excessive sweating, an increase risk of osteoporosis and hip fracture and many other side effects.

Hepatotoxicity due to hypervitaminosis A has been described for years and include alterations in liver profile, cholestasis, non-cirrhotic portal hypertension, chronic hepatitis and cirrhosis [110,111,112,113]. Hepatotoxicity at therapeutic doses has also been described. A case of severe hepatotoxicity associated with habitual daily ingestion of 25,000 IU of vitamin A bought as an over-the-counter DS was reported by Kowalski [114]. In addition, vitamin A tolerance can be reduced in patients with regular alcohol consumption [115]. Toxicity of vitamin A is belived to be a dose-dependent effect of retinoids on hepatic stellate cells. A study by Nollevaux showed a statistically significant correlation between the volumen density of total fibrosis and volumen density of total α-smooth muscle actin-immunolabelled cells. Moreover, histology assessment together with clinical data indicated a strong correlation between volumen density of perisinusoidal fibrosis and the daily dose of vitamin A intake [116].

We found eighteen reports with 58 cases of vitamin A containing DS related hepatotoxicity [110,111,114,116,117,118,119,120,121,122,123,124,125,126,127,128,129,130]. A rare case of cholestatic liver injury without fibrosis with pathological features of vitamin A accumulation in the liver biopsy has also been described, however the patient was taking Herbalife products, therefore it cannot be excluded the presence of hepatotoxicity of other components of Herbalife products together with vitamin A intoxication [111]. Other individual risk factors have been described such as pre-existing liver disease, co-medication with other potentially hepatotoxic drugs, and younger age [109,131].

## 3. Miscellaneous

### 3.1. Ma Huang Extract

Ephedra, also known as ma huang, is a medicinal preparation from the plant *Ephedra sinica* and is widely used as a weight-loss product by millions of people. Analysis of safety data from 50 clinical trial revealed that ma huang are associated with many adverse event related to this product (psychiatric, gastrointestinal, cardiovascular and cerebrovascular) [132].

Liver injury associated with ma huang has also been reported. In an analysis at four transplant center, 10 patients with severe liver injury were associated with the intake of DS for weight loss containing ma huang (Xenadrine, Excelerator, Metabolife 356, Thermolite, BetaLin, Thermo diet stack and Hydroxycut) [74]. Hycroxycut-induced liver injury cases has been described above. Time to onset was approximately 6 weeks or more. Liver profiles showed high level of transaminases and coagulopathy. The patients presented hepatic encephalopathy from grade 1 to 4 and three patients required liver transplantations, while the remaining 7 recovered without residual lesions.

### 3.2. Garcinia Cambogia

*Garcinia cambogia* is a plant-based supplement widly promoted for weight loss. It has been implicated in hepatotoxicity in patients taking Hydroxycut, wich contains a variety of ingredients, including *Garcinia cambogia*, as mentioned above. The use of *Garcinia cambogia* alone has also been implicated in cases of hepatotoxicity. A 52-year-old female needed an orthotopic liver transplant after taking *Garcinia cambogia* (USA Nutra Labs) 1000 mg (2 capsules/daily) during 15 days for weight loss [133]. Another case occurred in a 42-year-old female after taking pure *Garcinia cambogia* during one week also for weight loss. This patient had very high trasaminases level (ALT 70 xULN and AST 45 xULN) and coagulopathy (INR 1.3). After several days, the patient recovered and was discharged [134]. In both cases other ethiologies of liver injury were ruled out.

## 4. Discussion

Dietary supplements are regulated as food and not subjected to the same pre and postmarketing requirements for safety or efficacy as drugs do. However, published reports of DSILI are rising in parallel with the increasing popularity of herbal and DS in western countries. DSILI has been recognized, in some instances with cases of positive reexposure (grean tea extracts). However, the causality assessment in hepatotoxicity has limitations as there is an absence of diagnostic biomarkers. Furthermore, the attribution of causality is performed through an exhaustive interview with the patient, asking for the cronology of intake of drugs and HDS, and exclusion of alternative causes. For DS, reaching a consistent hepatotoxicity diagnosis is even more difficult as patients do not generally perceive them as harmful and therefore do not always inform about DS intake when being interviewed for an episode of liver damage. Furthermore, the use of DS may not always be regular. In addition, the ingredients in DS compounds can vary considerably and are not always adequately reflected in the product label. It is for that, some authors have analyzed some of the published reports and have profoundly criticized these case reports, the method of causality assessment used as well as the need for an update in regulations [63,104,135,136]. Despite the fact that some of the reported DSILI cases could be erroneously diagnosed, the great amount of data in the literature points to an important problem of health related to the consumption of DS.

The different forms of DSILI considered in this review can be differentiated into two groups based on their characteristic phenotypes: AAS and the remaining DSILI cases. While AAS hepatotoxicity typically produce high values of TB with low levels of transaminases, and no cases of ALF described to date [10,11,23,24,25,26], liver injury due to the remaining DS has typically a hepatocellular pattern of liver damage with very high transaminase levels [8,47,49,64,65,66,97,103] and a high number of cases with ALF [53,60,62,65,70,71,72,73,74,78,80,92,101,102]. Hence, DSILI constitutes an important cause of ALF in transplantation centers [73,74]. The clinical presentation, liver profile and outcome of DSILI (excluding AAS) coincide with that of herbal hepatotoxicity, while idiosyncratic hepatotoxicity due to conventional medication have a minor percentage of fatal cases and lower levels of transaminases compared to DSILI and lower levels of bilirubin compared to AAS liver injury [10,11].

In summary, given the unproven efficacy and the high number of cases of liver injury due to DS in addition to the severity with risk of acute liver failure and subsequent death or transplantation, stricter regulations for commercialization and sale of these products are required by competent health authorities. A joint effort among clinicians and health authorities in identifying new hepatotoxicity cases and to educate the general population on the risks of DS consumption is needed.

## Figures and Tables

**Table 1:** Tabular compilation of dietary supplements related to liver injury.

Dietary Supplement *	Citations	Number of Cases	Constituents **	Marketed Properties	Type of Liver Injury	Regulatory Measures
***Camellia sinensis*** **(green tea)** Euforia Exolise Onshido SlimQuick X-elles cha verde Curr Camilina-Arkocápsulas Lipolyz	García-Cortes *et al.*, 2008 [8]	3	Powdered leaves, hydroalcoholic and aqueus extracts and infusions from green tea leaves	Weight loss Stop hair loss	Acute hepatocellular injury	Hydroalcoholic extract of camellia Sinensis “EXOLISE” withdrawn from European market
	Navarro *et al.*, 2014 [10]	8				
	Gavilan *et al.*, 1999 [41]	1				
	Pillukat *et al.*, 2014 [42]	1				
	Molinari *et al.*, 2006 [43]	1				
	Verhelst *et al.*, 2009 [44]	1				
	Patel *et al.*, 2013 [45]	1				
	Lorenzo-Almorós *et al.*, 2015 [46]	1				
	Bjornsson *et al.*, 2007 [47]	5				
	Abu el Wafa *et al.*, 2005 [48]	1				
	Garcia-Moran *et al.*, 2004 [49]	1				
	Dueñas *et al.*, 2004 [50]	1				
	Thiolet *et al.*, 2002 [51]	1				
	Weinstein *et al.*, 2012 [52]	1				
	Sarma *et al.*, 2008 [53]	34				
	Mazzanti *et al.*, 2009 [54]	2				
	Gloro *et al.*, 2005 [60]	1				
	Pedros *et al.*, 2003 [61]	4				
	Seddik *et al.*, 2001 [137]	1				
	Vial *et al.*, 2003 [138]	1				
	Peyrin-Biroulet *et al.*, 2004 [139]	1				
	Mathieu *et al.*, 2005 [140]	1				
	Bonkovsky *et al.*, 2006 [141]	1				
	Javaid *et al.*, 2006 [142]	1				
	Jimemez-Saenz *et al.*, 2006 [143]	1				
	Rohde *et al.*, 2011 [144]	1				
	Martinez-Sierra *et al.*, 2006 [145]	1				
	Federico *et al.*, 2007 [146]	2				
	Prieto de Paula *et al.*, 2008 [147]	1				
	Bergman *et al.*, 2009 [148]	1				
	McDonnell *et al.*, 2009 [149]	1				
	Amariles *et al.*, 2009 [150]	1				
	Jiménez-Encarnacion *et al.*, 2012 [151]	1				
	Gallo *et al.*, 2013 [152]	1				
	Whislett *et al.*, 2014 [153]	1				
	Arzenton *et al.*, 2014 [154]	1				
	Fernandez *et al.*, 2014 [155]	3				
	Dela Cruz *et al.*, 2014 [156]	1				
	Van straelen *et al.*, 2008 [157]	1				
**Linoleic acid**	Ramos *et al.*, 2009 [64]	1	Polyunsaturated omega-6 fatty acid	Reduction in body fat mass	Hepatocellular	
	Nortadas *et al.*, 2012 [65]	1				
	Bilal *et al.*, 2015 [66]	1				
**Usnic acid** Lipokinetix, UCP-1 Lipolyz	Yellapu *et al.*, 2011 [62]	1	Usnic acid Norephedrine (phenylpropanolamine-PPA), caffeine, yohimbine, diiodothyronine, sodium usniate	Weight loss	Hepatocellular Acute hepatitis	FDA warning and withdrawn from the market
	Favreau *et al.*, 2002 [70]	7				
	Durazo *et al.*, 2004 [71]	1				
	Sanchez *et al.*, 2006 [72]	2				
	Estes *et al.*, 2003 [73]	2				
	Neff *et al.*, 2004 [74]	2				
	Hsu *et al.*, 2005 [75]	3				
**Vitamin A Retinol** Plethoryl	Ramanathan *et al.*, 2010 [111]	1				
	Becker *et al.*, 2007 [112]	1				
	Croquet *et al.*, 2000 [113]	1				
	Kowalski *et al.*, 1994 [114]	1				
	Geubel *et al.*, 1991 [110] and Nollevaux *et al.*, 2006 [116]	41				
	Muenter *et al.*, 1971 [117]	1	Retinoids	Immunostimulant, prevention of night blindness, acne	Chronic hepatitis, cirrhosis, cholestasis, non-cirrhotic portal hypertension	
	Russell *et al.*, 1974 [118] and Jaques *et al.*, 1979 [119]	1				
	Herbert *et al.*, 1981 [120]	1				
	Farris *et al.*, 1982 [121]	1				
	Weber *et al.*, 1982 [122]	1				
	Hatoff *et al.*, 1982 [123]	1				
	Park *et al.*, 1985 [124]	1				
	Inkeles *et al.*, 1986 [125]	1				
	Vincent *et al.*, 1986 [126]	1				
	Witzleben *et al.*, 1984 [127]	1				
	Minuk *et al.*, 1988 [128]	3				
	Jorens *et al.*, 1992 [129]	1				
	Cheruvattath *et al*., 2006 [130]	1				
Herbalife combinations	Navarro *et al.*, 2014 [10]	4	Numerous products with changes among countries	Weight loss nutritional support, well being	Acute hepatitis, cholestasis, cholestatic hepatitis, cirrhosis, ALF	
	Schoepfer *et al.*, 2007 [78]	10				
	Stickel *et al.*, 2009 [79]	2				
	Elinav *et al.*, 2007 [80]	12				
	Duque *et al.*, 2007 [81], Manso *et al.*, 2008 [82] and Manso *et al.*, 2011 [83]	20				
	Chao *et al.*, 2008 [84]	1				
	Johansson *et al.*, 2010 [85]	5				
	Chen *et al.*, 2010 [86]	2				
	Mengual-Moreno *et al.*, 2015 [87]	2				
	Ramanathan *et al.*, 2010 [111]	1				
Hydroxycut	Navarro *et al.*, 2014 [10]	5	*Camellia sinensis* Ma huang (ephedra) *Gymnema sylvestre Amorphophallus Konjac Paullinia cupana Garcinia cambogia* Caffeine a-Lipoic acid l-Carnitine Calcium Potassium Chromium	Weight loss	Hepatocellular pattern. Acute hepatitis, cholestasis, ALF, AIH	FDA warning
	Neff *et al.*, 2004 [74]	2				
	Chen *et al.*, 2010 [86]	1				
	Stevens *et al.*, 2005 [88]	2				
	Jones *et al.*, 2007 [89]	1				
	Dara *et al.*, 2008 [90]	2				
	Shim *et al.*, 2009 [91]	1				
	Fong *et al.*, 2010 [92]	8				
	Sharma *et al.*, 2010 [93]	1				
	Kaswala *et al.*, 2014 [94]	1				
	Araujo *et al.*, 2015 [97]	1				
	Laczek *et al.*, 2008 [98]	3				
	Haimowitz *et al.*, 2015 [99]	1				
Oxy ELITE Pro	Navarro *et al.*, 2014 [10]	1	Multiingredient. 1,3-Dimethylamylamine (DMAA) Aegeline (*N*-[2-hydroxy-2(4-methoxyphenyl) ethyl]-3-phenyl-2-propenamide)	Weight loss Muscle building	Acute hepatitis, ALF	FDA warning and withdrawn from the market
	Foley *et al.*, 2014 [101]	7				
	Roytman *et al.*, 2014 [102]	8				
	Johnston *et al.*, 2015 [103]	36				
	Klontz *et al.*, 2015 [105]	55 including the 36 from Johnston				
**Illicit androgenic anabolic steroids:** Celtic dragon Episdrol Epistane SUS500 Uprizing 2.0	Navarro *et al.*, 2014 [10]	45	Androstenedione Dehidroepiandrosterone Desoxymethyltestosterone Maasdrol Stanozolol Methasterone Methylepithiostanol Superdrol	Body-building, improving fitness and exercise performance	Hepatocellular hepatitis, cholestatic hepatitis, hepatocellular adenoma, hepatocellular carcinoma, peliosis hepatitis, focal nodular hyperplasia	Episdrol and Epistane were withdrawn from the Spanish market, Uprizing 2.0 (superdrol): FDA warning and recall of the product
	Robles-Diaz *et al.*, 2015 [11]	25				
	Singh *et al.*, 2009 [22]	3				
	Timcheh-Hariri *et al.*, 2012 [23]	20				
	Kafrouni *et al.*, 2007 [24]	2				
	Shah *et al.*, 2008 [25]	5				
	Krishnan *et al.*, 2009 [26]	3				
	Schwingel *et al.*, 2015 [27]	23				
	Turani *et al.*, 1983 [28]	11				
	Choi *et al.*, 2009 [29]	1				
	Karasawa *et al.*, 1979 [30]	5				
	Patil *et al.*, 2007 [31]	1				
	Agbenyefia *et al.*, 2014 [32]	1				
	Hymel *et al.*, 2013 [33]	1				
	Brazeau *et al.*, 2015 [35]	1				
	Sánchez Osorio *et al.*, 2008 [36]	1				
	Vilella *et al.*, 2013 [37]	1				
	El Sherrif *et al.*, 2013 [38]	2				
	Wingert *et al.*, 2010 [158]	1				
	Luciano *et al.*, 2014 [159]	1				
**Ma huang (ephedra)** Xenadrine, Excelerator, Metabolife 356, Thermolite, BetaLin, Thermo diet stack, Hydroxycut	Neff *et al.*, 2004 [74]	10 (including 2 cases of Hydroxycut with Ma Huang reported above)	MA HUANG (ephedra)	Weight loss	Hepatocellular ALF	
***Garcinia cambogia***	Corey [133]	1	*Garcinia cambogia*	Weight loss	Hepatocellular ALF	
	Melendez-Rosado [134]	1				

ALF: acute liver failure. * Dietary supplements (DS) listed are single ingredient (vitamin A, *Garcinia cambogia*), single ingredient with examples of marketed DS containing, among others, this single ingredient (Usnic acid: Lipokinetix, UCP-1), marketed DS with several ingredients (Hydroxycut, Herbalife combinations); ** constituents are some of the ingredients contained in the DS in the same row. Not all the ingredients are present in each DS. The formulation of many DS has changed over the years. DS in bold correspond to single ingredients; the remaining DS correspond to brand names.

## Abbreviations

AAS: anabolic androgenic steroids
AEMPS: Spanish Drug and Sanitary Products Agency
ALF: acute liver failure
ALP: alkalin phosphatase
ALT: alanine aminotransferase
AST: aspartate aminotransferase
ATC: Anatomical Therapeutic Chemical
CLA: conjugated linoleic acid
DS: dietary supplements
DILI: drug-induced liver injury
DILIN: drug-induced liver injury network
DMAA: dimethylamylamine
DSHEA: Dietary Supplement Health and Education Act
DSILI: dietary supplements-induced liver injury
FDA: Food and Drug Administration
HDS: herbal and dietary supplements
HDS-DILI: herbal and dietary supplement-induced liver injury
INR: international normalized ratio
NAFLD: non-alcoholic fatty liver disease
OEP: OxyELITE Pro
RUCAM: Roussel Uclaf Causality Assessment Method
TB: total bilirubin
UK: United Kingdom
US: United States
ULN: upper limit of normal
